# Endogenous attention is invariant to sequential effects in performance around the visual field

**DOI:** 10.64898/2026.07.18.739376

**Published:** 2026-09-30

**Authors:** Hsing-Hao Lee, Marisa Carrasco

## Abstract

Visual perception is shaped by both current sensory inputs and contextual information across space and time, including the perception of preceding trials. Covert spatial attention selects and prioritizes relevant sensory information. Endogenous attention is voluntary, goal-driven, and flexible. However, it cannot alleviate visual polar-angle asymmetries, specifically, the horizontal-vertical anisotropy and the vertical meridian asymmetry. Although previous studies reported sequential effects whereby attention interacts with response repetitions, it is unknown whether and how endogenous attentional modulation of performance varies as a function of target location and trial history. Here, we reanalyzed data from three published studies of endogenous attention in orientation discrimination tasks, to (1) assess typical sequential effects on response, in which response to the current trial is biased toward the previous one, and (2) examine whether sequential effects modulate performance across visual field locations, along four dimensions: (1) target location, (2) target feature, (3) attention condition, and (4) the correctness of the preceding ( *n –* 1) trial. First, we observed typical sequential response repetition effects tied to the repeated location and feature properties of the target. Second, whereas attention robustly modulated performance, none of the three studies showed evidence of sequential attention effects on performance across any of the four dimensions, nor did trial history reveal any attention-by-location interactions. Together, these findings provide compelling evidence that visual polar-angle asymmetries are resistant to endogenous attention, and that even sequential effects–involving bottom up and top-down processes–do not mitigate these performance asymmetries.

## Full Text Availability

The license terms selected by the author(s) for this preprint version do not permit archiving in PMC. The full text is available from the preprint server.

